# Five-year change in body mass index category of childhood and the establishment of an obesity prediction model

**DOI:** 10.1038/s41598-020-67366-y

**Published:** 2020-07-16

**Authors:** Yuelin Sun, Yufang Xing, Junfeng Liu, Xiaoxia Zhang, Jingyu Liu, Zhaoxia Wang, Jingyang Bi, Xianghe Ping, Qiqiang Shen, Zhouqiao Zhao, Jinjie Xu

**Affiliations:** 1 Yantai Center for Disease Control and Prevention, 17 Fuhou Road, Laishan District, YanTai, 264003 Shandong China; 2 School of Mathematics and Information Sciences, Yantai University, YanTai, 264005 China; 3 Department of Atmospheric and Oceanic Sciences, School of Physics, Peking University, Beijing, 100871 China

**Keywords:** Evolution, Health care, Risk factors

## Abstract

The prevalence of childhood obesity in China has recently become increasingly severe, and intervention measures are needed to stop its growth. Currently, there is a lack of assessment and prediction methods for childhood obesity. We develop a predictive model that uses currently measured predictors [gender, age, urban/rural, height and body mass index (BMI)] to quantify children’s probabilities of belonging to one of four BMI category 5 years later and identify the high-risk group for possible intervention. A total of 88,980 students underwent a routine standard physical examination and were reexamined 5 years later to complete the study. The full model shows that boys, urban residence and height have positive effects and that age has a negative effect on transition to the overweight or obese category along with significant BMI effects. Our model correctly predicts BMI categories 5 years later for 70% of the students. From 2018 to 2023, the prevalence of obesity in rural boys and girls is expected to increase by 4% and 2%, respectively, while that in urban boys and girls is expected to remain unchanged. Predictive models help us assess the severity of childhood obesity and take targeted interventions and treatments to prevent it.

## Background

With the rapid growth of the social economy and lifestyle changes, childhood overweight and/or obesity prevalence is increasing globally at an alarming pace, which has greatly increased public health concerns.^1,2^ The prevalence of childhood obesity in China has increased rapidly in the past two decades. According to statistics on overweight in Chinese children and adults, from 1985 to 2014, the overweight rate among schoolchildren (over 7 years old) increased from 2.1 to 12.2%, and the obesity rate increased from 0.5 to 7.3%. Without effective interventions, the overweight and obesity rates will reach 28.0% by 2030, with a total of 49.48 million overweight and/or obese children.^3^ Childhood obesity is an important predictor of several adulthood chronic diseases, such as hypertension, type 2 diabetes mellitus, obstructive sleep apnea, and psychological and behavioral problems.^4^ It has been shown that 72% and 63% of children in kindergarten and the following three years of school (between ages 11 and 14 years old), respectively, are obese.^5^ Thus, BMI trajectories from childhood to adulthood could improve our understanding of the evolution of the prevalence of childhood obesity. In addition, estimating age-specific morbidity rates would facilitate the development future intervention strategies.^6,7^ Moreover, if we can target children at high risk of future or continuing obesity based on a set of significant factors, personalized interventions would be feasible to prevent and/or decrease the incidence of obesity. Although a number of studies on the prevalence of childhood obesity are available,^4,5,8^ few studies have quantified the joint impacts of recognized predictors of obesity after a specified number of years (the time window for implementing an intervention). Therefore, we conducted a 5-year follow-up study on primary school students in Yantai, China, to establish a reliable predictive model to quantify the probability of each student being in a certain BMI category after 5 years. Our study mainly aimed to (1) study the transition rates (from the beginning to the end of the study) across four BMI categories; (2) establish an efficient overweight/obesity probability model with improved predictive performance (e.g., compared to that using only the BMI categories form the beginning of the study); and (3) identify the high-risk group through the definition of a joint high-risk domain based on the significant predictors.

## Methods

### Data collection and processing

The human research ethics committee at our institute (Yantai Center for Disease Control and Prevention) approved this study after confirming that all the procedures, including the anthropometric procedures and data collection, were performed in accordance with the relevant guidelines and regulations. Students’ written informed consent was obtained from parents, legal guardians, or both. The participants of the current cohort were recruited randomly from primary and junior high schools (in Yantai) in September 2013 during a physical examination conducted by our institute. All participants were followed up five years later (in September 2018). A total of 96,264 individuals were enrolled when the study began, and 88,980 completed the study (with a small proportion missing completely at random due to irrelevant issues).

Description of anthropometric measures: All researchers participated in training on anthropometric procedures and data collection, and all instruments were calibrated. The children were asked to take off thick clothing, shoes and socks during the measurement process, and their weight and height were measured to the nearest 0.1 cm and 0.1 kg, respectively. In addition, the children’s name, gender, date of birth, grade, and area or residence (urban or rural) were collected.

Body mass index (BMI) is calculated as weight (kg)/height (m)^2^; the evaluation criteria for underweight, normal, overweight and obese vary for different genders and age groups per Chinese national criteria specifically designed to provide a comprehensive evaluation standard for school-aged children and adolescent development.^9,10^ The large sample size is sufficient to accurately estimate the small group of parameters [point estimate and 95% confidence interval (CI)] and test the significance of each predictor.

### Statistical analysis

The mean height and BMI in the population show different increasing trends with age. The increases also depend on both gender and district. We first use bar charts to present the exploratory analysis results of the distributions of follow-up BMI categories by making marginal comparisons between groups (e.g., boys and girls). Our primary analysis uses a baseline-category logit model to quantify the final BMI category probabilities based on the set of predictors measured at the beginning of the study. The prediction of the BMI category (in 2023) can be made based on the new data, and the high-risk (high probability of obesity) group can be identified. Data analysis and model fitting are implemented by using R 3.5.0. and SAS 9.2.

### Model fitting

We apply the following baseline-category logit model:

1 $$\log \frac{{p_{j} \left( x \right)}}{{p_{2} \left( x \right)}} = \beta _{{j,0}} + \sum_{i} {\beta _{{j,i}} } \cdot x_{i}$$

Here the baseline category is *J* = 2 (“normal”), and three other categories (*j* = 1, 3, 4) represent “underweight”, “overweight” and “obese” with the following category probabilities:

2 $$p_{2} \left( x \right) = \frac{1}{{1 + \sum_{{j \in \{ 1,3,4\} }} {e^{{\beta _{{j,0}} + \sum_{i} {\beta _{{j,i}} } \cdot x_{i} }} } }}, \quad p_{j} \left( x \right) = \frac{{e^{{\beta _{{j,0}} + \sum_{i} {\beta _{{j,i}} } \cdot x_{i} }} }}{{1 + \sum_{{k \in \{ 1,3,4\} }} {e^{{^{{\beta _{{k,0}} + \sum_{i} {\beta _{{k,i}} } \cdot x_{i} }} }} } }}.$$

**Figure 1:**
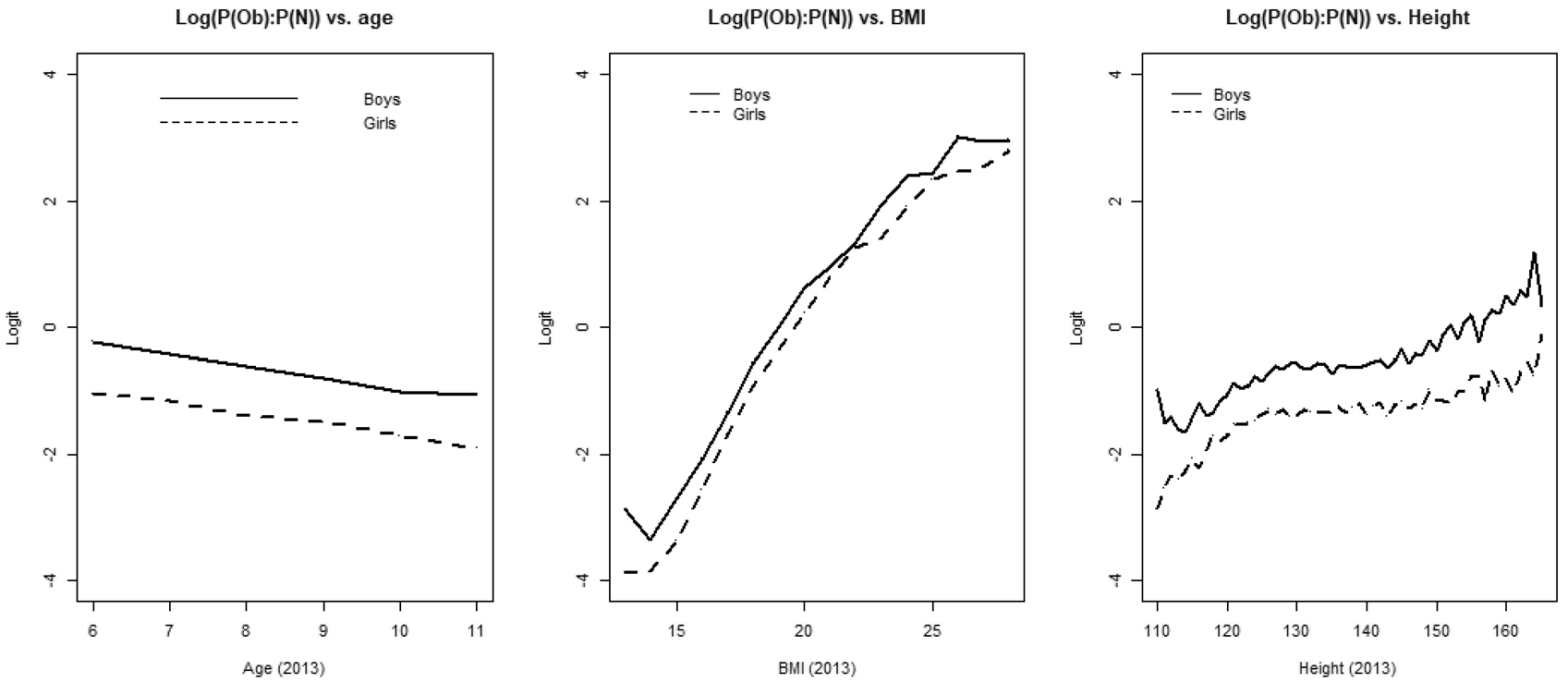
The empirical log(probability ratio [obese:normal]) versus age and BMI.

Here predictor *x* is a vector of variables (*x*_*i*_, *i* = 1…,5), i.e., gender, district, age, height and BMI13 [weight is omitted due to the relationship of BMI = weight(kg)/height(m)^2^]. We calculate the empirical probabilities of being in the “obese” and “normal” categories for each age group, and the log(probability ratio) profiles (Fig. 1) indicate no interactions (between age, BMI, height and gender); similar observations are found for the district variable. Thus, a linear age effect in Eq. (1) applies.

The interactions found in this preliminary study are either nonsignificant or of little interest to us and are excluded from the model. The large sample size is sufficient for our parameter significance test given the small set of predictors.

To efficiently estimate the parameters, we employ the separate maximum likelihood estimation (SMLE) approach by fitting a separate logistic model to each subpopulation (response (*Y*) = {*j*, 2} in Eq. (2)), where $p_{j}^{*} \left( x \right)$ represents the event (Y = *j*) probability with parameter values identical to those in Eqs. (1–2), i.e.,

3 $$\log \frac{{p_{j}^{*} \left( x \right)}}{{1 - p_{j}^{*} \left( x \right)}} = \beta _{{j,0}} + \sum_{i} {\beta _{{j,i}} } \cdot x_{i} \quad (j = 1,3,4)$$

ROC curves, the 5-year-later multi-category prediction outcome classification table and comparison with other model fitting algorithms (e.g., global maximum likelihood estimation (GMLE) using Newton–Raphson iteration) are made to evaluate our approach. The high-risk group is determined to enable possible intervention. We restrict BMI13 to be within [10,35] (*n* = 88,879) to avoid program running errors.

## Results

### Descriptive statistics

The general characteristics of the children at baseline are listed in Table 1. The initial sample includes 96,264 children (49,220 boys and 47,044 girls aged 6–11 at 2013). Students of Han nationality account for 99.86% of the sample, while those of other nationalities account for 0.14%. A total of 55.66% of the sample comes from urban areas.

**Table 1:** General characteristics of children at baseline by gender (2013).

Category	Boys (n, %)	Girls (n, %)	Overall (n, %)
Age (years)			
6	6,935 (14)	7,062 (15)	13,997 (15)
7	10,620 (22)	10,091 (21)	20,711 (22)
8	11,317 (23)	10,612 (23)	21,929 (23)
9	10,883 (22)	10,452 (22)	21,335 (22)
10	6,507 (13)	6,183 (13)	12,690 (13)
11	2,958 (6)	2,644 (6)	5,602 (6)
Ethnicity			
Han	49,151 (99.9)	46,978 (99.9)	96,129 (99.9)
Others	69 (0.1)	66 (0.1)	135 (0.1)
District			
Urban	27,694 (56)	25,885 (55)	53,579 (56)
Rural	21,526 (44)	21,159 (45)	42,685 (44)
Overall	49,220 (51)	47,044 (49)	96,264 (100)

### Cross-category analysis

**Figure 2:**
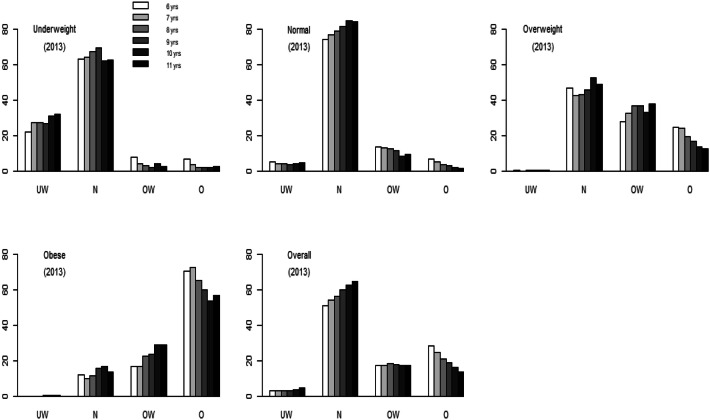
BMI category (2018) proportions (stratified by age in 2013).

Cross-category changes (from 2013 to 2018) are summarized in Table 2. In 2013, the most prevalent BMI category among the children is normal weight (55%), followed by obese (25%), overweight (17%) and underweight (3%). The prevalence of obesity decreases significantly from 2013 to 2018 (95% CI 0.02, 0.03). A total of 3%, 4% and 20% of the children in the underweight, normal and overweight groups in 2013, respectively, transition to the obese group in 2018. Sixty-five percent of the 2013 obese group is still the obese group in 2018. Seventy-three percent of the 2018 obese group had been obese in 2013. Among the children in the obese group in 2013, 13% and 22% transition into the normal and overweight groups in 2018, respectively. The prevalence rates are plotted in Fig. 2 (stratified and grouped by age and BMI13 category). The majority of the normal-weight or obese students in 2018 are in the same category that they were in in 2013. Boys in all four BMI13 categories are more likely than girls to be obese in 2018 [*p* < 0.001, 95% CI of the odds ratio (OR) 1.66, 3.90; 1.66, 1.99; 1.28, 1.50; and 1.46, 1.64, respectively]. Girls from the 2013 obese group are more likely be overweight in 2018 than boys (*p* < 0.001, 95% CI of the OR 1.12, 1.28). Urban students are more likely than rural students be obese in 2018 only among those students who had been in the obese group in 2013 (*p* < 0.001, 95% CI of the OR 1.08, 1.21). The obesity prevalence rate generally decreases as age (in 2013) increases (Fig. 1).

**Table 2:** Matrix distributions of categorical body mass index (from 2013 to 2018).

Category	2018				
	Underweight *n* (%)	Normal *n* (%)	Overweight *n* (%)	Obesity *n* (%)	Overall *n* (%)
2013					
Underweight	801 (27)	1,928 (66)	116 (4)	95 (3)	2,940 (3)
	*605 *(*21*)	*2,155 *(*74*)	*117 *(*4*)	*27* (*1*)	
Normal	2,140 (4)	38,868 (79)	5,891 (12)	2,025 (4)	48,924 (55)
	*2,334* (*5*)	*37,259* (*76*)	*6,521* (*13*)	*2,809* (*6*)	
Overweight	74 (0.5)	6,961 (46)	5,154 (34)	3,089 (20)	15,278 (17)
	*85* (*0.6*)	*7,812* (*51*)	*4,217* (*28*)	*3,164* (*21*)	
Obesity	77 (0.4)	2,843 (13)	4,732 (22)	14,186 (65)	21,838 (25)
	*11* (*0.05*)	*3,350* (*15*)	*5,903* (*27*)	*12,509* (*57*)	
Overall	3,092 (3)	50,600 (57)	15,893 (18)	19,395 (22)	88,980 (100)
	*3,035* (*3*)	*50,577* (*57*)	*16,758* (*19*)	*18,509* (*21*)	
Boys					
Underweight	353 (29)	752 (61)	70 (6)	61 (5)	1,236 (3)
	*276* (*23*)	*867* (*71*)	*62* (*5*)	*15* (*1*)	
Normal	1,067 (5)	17,251 (75)	3,326 (15)	1,234 (5)	22,878 (50)
	*1,159* (*5*)	*16,515* (*72*)	*3,586* (*16*)	*1,617* (*7*)	
Overweight	43 (0.5)	3,387 (41)	3,000 (36)	1,876 (23)	8,306 (18)
	*46* (*0.6*)	*3,833* (*46*)	*2,500* (*30*)	*1,927* (*23*)	
Obesity	43 (0.3)	1,348 (10)	2,681 (20)	9,048 (69)	13,120 (29)
	*5* (*0.04*)	*1,521* (*12*)	*3,477* (*27*)	*8,071* (*62*)	
Overall	1,506 (3)	22,738 (50)	9,077 (20)	12,219 (27)	45,540 (100)
	*1,487* (*3*)	*22,737* (*50*)	*9,625* (*21*)	*11,630* (*26*)	
Girls					
Underweight	448 (26)	1,176 (69)	46 (3)	34 (2)	1,704 (4)
	*329* (*20*)	*1,288* (*76*)	*55 *(*3*)	*12* (*1*)	
Normal	1,073 (4)	21,617 (83)	2,565 (10)	791 (3)	26,046 (60)
	*1,175* (*5*)	*20,744* (*80*)	*2,935* (*11*)	*1,192* (*5*)	
Overweight	31 (0.4)	3,574 (51)	2,154 (31)	1,213 (17)	6,972 (16)
	*39* (*1*)	*3,980* (*57*)	*1,717* (*25*)	*1,237* (*18*)	
Obesity	34 (0.4)	1,495 (17)	2,051 (24)	5,138 (59)	8,718 (20)
	*5* (*0.1*)	*1,828* (*21*)	*2,426* (*28*)	*4,438* (*51*)	
Overall	1,586 (4)	27,862 (64)	6,816 (16)	7,176 (17)	43,440 (100)
	*1,548* (*4*)	*27,840* (*64*)	*7,133* (*16*)	*6,878* (*16*)	
Urban					
Underweight	388 (29)	859 (64)	60 (4)	41 (3)	1,348 (3)
	*286 *(*22*)	*973* (*73*)	*58 *(*4*)	*13* (*1*)	
Normal	1,132 (4)	19,800 (79)	3,195 (13)	1,070 (4)	25,197 (52)
	*1,213* (*5*)	*18,819* (*75*)	*3,605* (*14*)	*1,560* (*6*)	
Overweight	39 (0.4)	3,894 (44)	3,067 (35)	1,795 (20)	8,795 (18)
	*51* (*1*)	*4,375* (*50*)	*2,506* (2*8*)	*1,864* (*21*)	
Obesity	34 (0.3)	1,432 (11)	2,949 (22)	9,004 (67)	13,419 (28)
	*7* (*0.1*)	*1,996* (*15*)	*3,730* (*27*)	*7,852* (*58*)	
Overall	1,593 (3)	25,985 (53)	9,271 (19)	11,910 (24)	48,759 (100)
	*1,557* (*3*)	*26,162* (*53*)	*9,899* (*20*)	*11,290* (*23*)	
Rural					
Underweight	413 (26)	1,069 (67)	56 (4)	54 (3)	1,592 (4)
	*319* (*20*)	*1,182* (*75*)	*59* (*4*)	*13* (*1*)	
Normal	1,008 (4)	19,068 (80)	2,696 (11)	955 (4)	23,727 (59)
	*1,121* (*5*)	*18,440* (*78*)	*2,916* (*12*)	*1,249* (*5*)	
Overweight	35 (0.5)	3,067 (47)	2,087 (32)	1,294 (20)	6,483 (16)
	*34 *(*1*)	*3,438* (*53*)	*1,711* (*26*)	*1,300* (*20*)	
Obesity	43 (0.5)	1,211 (15)	1,783 (22)	5,182 (63)	8,219 (21)
	*4* (*0.05*)	*1,354 *(*17*)	*2,174* (*27*)	*4,657* (*57*)	
Overall	1,499 (4)	24,415 (61)	6,622 (17)	7,485 (19)	40,021 (100)
	*1,478* (*4*)	*24,414* (*61*)	*6,859* (*17*)	*7,219* (*18*)	

Overweight and obesity are defined by References ^9,10^. The expected counts from fitting the full model using SMLE (Methods section) are given in italic font.

### Model-based analysis

**Figure 3:**
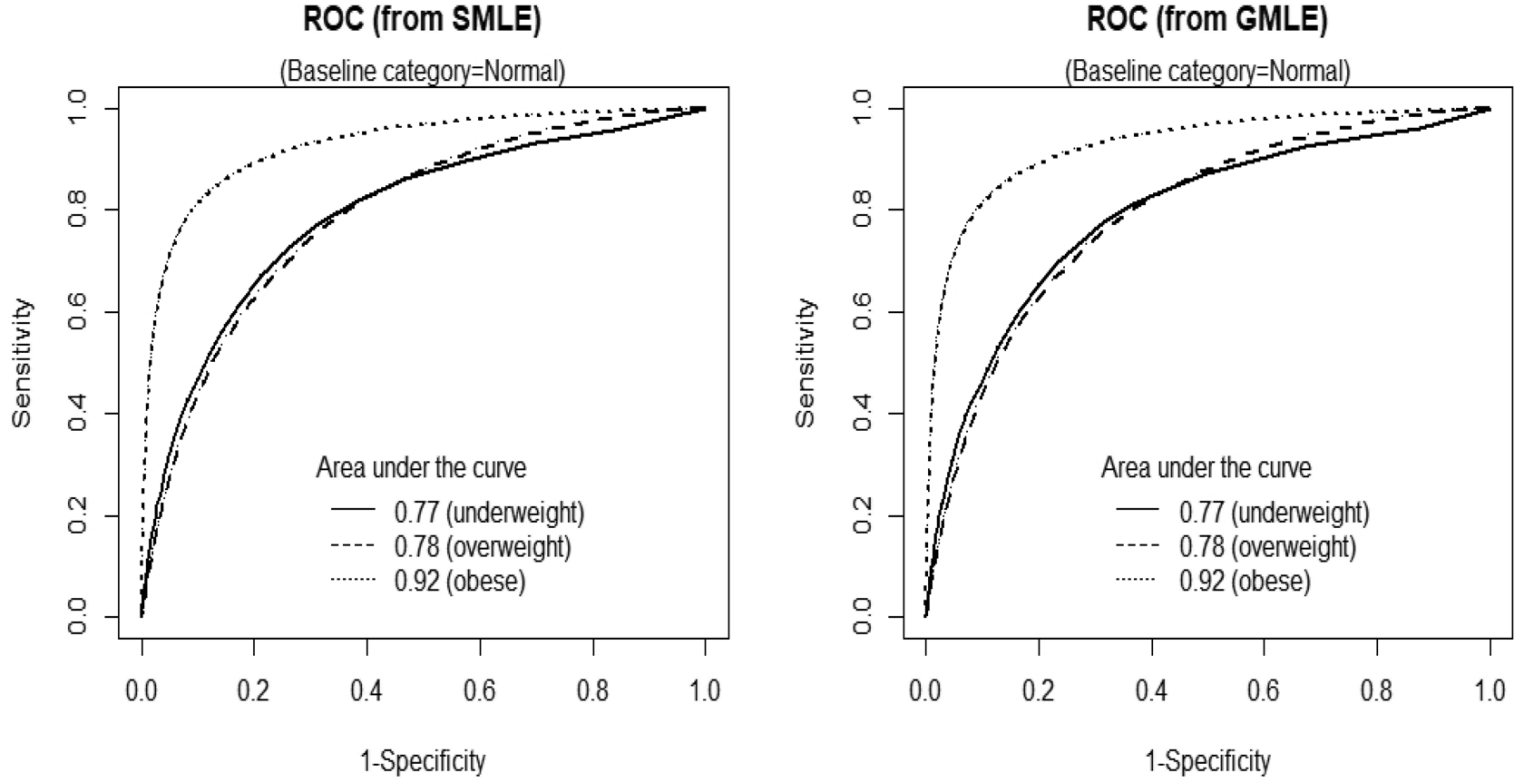
ROC curves from SMLE and GMLE model fitting approaches.

The SMLE parameter estimates are summarized in Table 3; all the predictors in Eq. (1) were significant (*p* < 0.05). Gender and district both have positive effects on the transition of students to the other three BMI categories from the “normal” category. Age has a positive (negative) effect on the transition of students to the “underweight” (“overweight” or “obese”) category from the “normal” category, and the effect of height is opposite to that of age. The linear BMI13 effect has opposite signs for the “underweight” versus “overweight” and “obese” categories. The ROC curves from the three logistic models have areas under the curve of 0.77, 0.78 and 0.92 (the left panel, Fig. 3). Height is also a significant predictor in addition to BMI13. The estimated probabilities of students being overweight and obese in the year 2023 using the recently collected 2018 physical examination data (*n* = 300,733) indicate that the obesity rates of boys and girls at all ages (6–11) in 2023 are higher than those in 2018. Specifically, among students aged 6 to 11, the obesity rates for boys in 2018 versus 2023 are 32 versus 34%, 27 versus 29%, 25 versus 27%, 23 versus 27%, 22 versus 26%, and 19 versus 24%, and the obesity rates for girls in 2013 versus 2018 are 22 versus 23%, 17 versus 19%, 15 versus 17%, 14 versus 16%, 13 versus 15%, and 9 versus 14%, respectively. The obesity rates among rural students (boys and girls) in 2023 appear to increase substantially compared to those of their 2018 peers (boys: 22–26%, girls: 14–16%). The overweight rates among urban students (boys and girls) and the overall sample (stratified by gender and district) show negligible changes from 2018 to 2023.

**Table 3:** Parameter estimates from fitting three separate logistic regressions.

	Pr(Underweight|UW,N)	Pr(Overweight|N,OW)	Pr(Obese|N,O)
Intercept			
SMLE	9.17 (8.42, 9.93)	− 7.57 (− 7.89, − 7.23)	− 10.20 (− 10.60, − 9.79)
GMLE	9.82 (9.10, 10.53)	− 7.89 (− 8.19, − 7.59)	− 11.04 (− 11.39, − 10.68)
Gender			
SMLE	0.43 (0.34, 0.51)	0.34 (0.30, 0.38)	0.39 (0.34, 0.44)
	**(1.40, 1.67)**	**(1.35, 1.46)**	**(1.40, 1.55)**
GMLE	0.41 (0.33, 0.48)	0.34 (0.30, 0.38)	0.40 (0.35, 0.44)
	**(1.40, 1.62)**	**(1.35, 1.46)**	**(1.42, 1.56)**
District			
SMLE	0.14 (0.06, 0.22)	0.12 (0.08, 0.17)	0.07 (0.01, 0.12)
	**(1.06, 1.25)**	**(1.08, 1.19)**	**(1.01, 1.13)**
GMLE	0.15 (0.07, 0.22)	0.10 (0.06, 0.14)	0.08 (0.03, 0.12)
	**(1.07, 1.25)**	**(1.07, 1.15)**	**(1.03, 1.13)**
Age			
SMLE	0.44 (0.39, 0.48)	− 0.48 (− 0.51, − 0.46)	− 0.97 (− 1.00, − 0.93)
	**(1.48, 1.62)**	**(0.60, 0.63)**	**(0.37, 0.39)**
GMLE	0.45 (0.40, 0.49)	− 0.46 (− 0.48, − 0.44)	− 1.04 (− 1.07, − 1.01)
	**(1.50, 1.63)**	**(0.62, 0.65)**	**(0.34, 0.37)**
Height			
SMLE	− 0.05 (− 0.05, − 0.04)	0.02 (0.02, 0.03)	0.03 (0.03, 0.04)
	**(0.95, 0.96)**	**(1.02, 1.03)**	**(1.03, 1.04)**
GMLE	− 0.05 (− 0.06, − 0.04)	0.02 (0.02, 0.02 )	0.03 (0.02, 0.04)
	**(0.94, 0.96)**	**(1.02, 1.02)**	**(1.03, 1.04)**
BMI13			
SMLE	− 0.64 (− 0.67, − 0.61)	0.43 (0.42, 0.44)	0.69 (0.68, 0.70)
	**(0.51, 0.54)**	**(1.52, 1.55)**	**(1.97, 2.01)**
GMLE	− 0.63 (− 0.66, − 0.61)	0.43 (0.42, 0.44)	0.76 (0.75, 0.77)
	**(0.52, 0.55)**	**(1.52, 1.55)**	**(2.11, 2.15)**

Pr(Underweight|UW,N), Pr(Overweight|N,OW) and Pr(Obese|N,O) represent the probabilities of “underweight”, “overweight” and “obese” regarding logistic regressions (Methods section). Point estimates with 95% confidence intervals (in parentheses) are compared between SMLE and GMLE approaches. Gender = 1 (male) or 0 (female). District = 1 (urban) or 0 (rural). BMI13 = BMI measured in 2013. The bold numbers are 95% confidence intervals for the adjusted odds ratios.

### Evaluating the SMLE approach

A bootstrap study provides the correlation structure among three sets of estimated regression parameters [Eq. (1)], where the true parameter values are those point estimates (SMLE) in Table 3 and the predictor (*X*) population is identical to that one from which the model is fitted (Table 3). The bootstrapped SML estimates have means highly close to the true values and variances highly consistent with the confidence intervals in Table 3. The correlation coefficients among these estimated 18 (3 × 6) parameters are useful for further inference (e.g., apply multiplicity adjustment to control false discovery). For instance, the estimated “age” and “height” coefficients are highly negatively correlated for each nutrition category [*j* = 1, 3, 4 in Eqs. (1–3)]:

$$\rho \left( {\hat{\beta }_{{1,3}} ,\hat{\beta }_{{1,4}} } \right) =-0.77, \quad \rho \left( {\hat{\beta }_{{3,3}} ,\hat{\beta }_{{3,4}} } \right) = - 0.75 \quad \text{and}\quad \rho \left( {\hat{\beta }_{{4,3}} ,\hat{\beta }_{{4,4}} } \right) = -0.73.$$

There are some other moderately correlated coefficient estimates. In comparison, SAS procedure Logistic employs GMLE (global maximal likelihood estimation) to fit models. The score test rejects the proportional odds model (*p* < 0.0001). For fitting the baseline-category logit model, SAS output (GMLE) highly agrees with SMLE for both point estimates and 95% CIs (Table 3) and SMLE (GMLE) has a log-likelihood of − 69,913 (− 69,806). ROC curves from GMLE (the right panel, Fig. 3) have under-the-curve areas equal to SMLE. SMLE appears to work efficiently without the need of complicated algorithms or expensive software packages.

### Model-based prediction

Based on Table 3, the predicted BMI category 5 years later is that with the largest probability cross *j* = {1:4} (a linear discriminant rule targeting the maximal element from {$x\beta _{1}$, 0, $x\beta _{3}$, $x\beta _{4}$}). A crude prediction by assigning all students to be “normal” in 2018 only has 57% (= 50,600/88,980, Table 2) of all students being correctly predicted. We are interested in comparing the prediction performance of the full model [Eqs. (1–2)] with that of Table 1 (only based on the BMI13 category). The full model correctly predicts the 5-year BMI category for 70% of students. The model-based prediction generally has a higher specificity (for predicting “overweight” and/or “obese”) than Table 1. Since the overall obesity (normal) prevalence rates naturally decreases (increases) from 2013 to 2018 (Table 2), the prediction of the prevalence of obesity (normal weight) using only the BMI13 category tends to inherently inflate (deflate) the sensitivity. To verify the prediction improvement, we randomly divide the data set into a training set and a testing set (fivefold cross-validation) 100 times. At each time, the fitted model from the training set is used to make BMI category predictions on the testing set. On average, 70% (SD  0.3%) of students are correctly predicted, the sensitivity means are (0.2, 94, 6, 68)% and the specificity means are (15, 71, 38, 69)%. These are very close to Table 4.

**Table 4:** Mode-based prediction outcome classification.

	Underweight^P^	Normal^P^	Overweight^P^	Obese^P^	% (sensitivity)
Underweight^O^	**6**	3,003	10	67	2% (26%)
Normal^O^	26	**47,789**	677	2,082	95% (77%)
Overweight^O^	1	11,246	**919**	3,703	6% (32%)
Obese^O^	5	5,473	772	**13,100**	68% (73%)
% (specificity)	16% (26%)	71% (79%)	39% (34%)	69% (65%)	**70%** (66%)

The left column (label ^O^) denotes the observed numbers. The top row (label ^P^) denotes the predicted numbers. The bold numbers represent the correctly predicted numbers. The correction rates from Table 2 are in the parentheses.

Based upon the preceding procedures, the identification of a high-obesity-risk group is feasible. The joint high-risk decision domain is developed based on the following crude ranges of various predictors: 6–11 (age), 110–170 (height (cm)) and 10–35 (BMI). For illustration purposes, given a threshold of 0.9, we calculate the obesity probability (Pr(O)) for each student recorded in 2018 (stratified by gender and district, age = 6–11).

**Figure 4:**
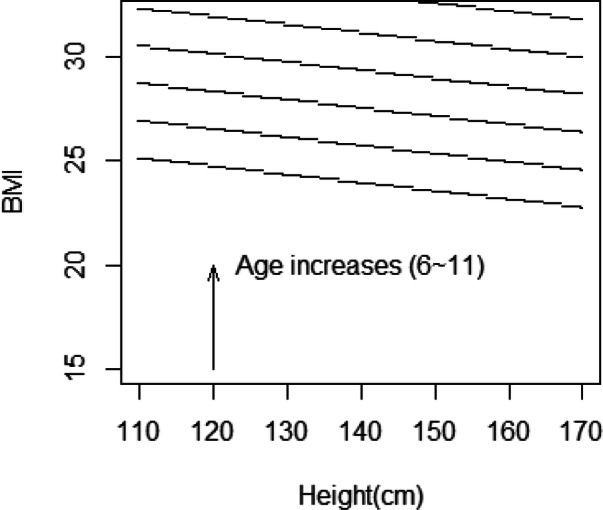
High-risk groups defined by height and BMI (urban boys, stratified by age).

Students with a Pr(O) > 0.9 would be included in the high-risk group with the joint (height, BMI) region (i.e., lower BMI boundary) calculated and plotted (e.g., for boys in the city) in Fig. 4. As age increases, the high-risk group boundary moves towards the upper-right corner.

## Discussion

Our study is an observational population cohort study. We develop a baseline-category logit model to quantify the probabilities of future BMI categories with the purpose of generating new evidence to reform policy based on the most effective ways to reduce childhood obesity. The high-risk group can be identified by BMI category prediction. The model is substantively accountable in predicting obesity incidence given the currently recognized set of factors. The estimated overweight and obese prevalence rates in 2023 indicate that the obesity rates of both boys and girls at all ages in 2023 appear to be substantially higher than those in 2018. The obesity rates among rural students in 2023 appear to increase substantially compared to those of their 2018 peers.

Our study shows that the probability of becoming obese decreases as age increases and that most high-risk students become high risk earlier (age ≤ 9) rather than later during childhood. Children who entered the study with a normal BMI were less likely to be obese 5 years later than those who entered with an overweight or obese BMI. Generally, a weight development trend is established in early childhood that lasts into adulthood, and it is better to prevent childhood obesity before its onset since transitioning to a normal weight from being obese is difficult, and persistent obesity is common.^11–13^ For example, two cohort studies in the United States showed that 65% of 5th-grade obese patients remained obese in 10th grade, 75% of 7-year-old obese patients were still obese at the age of 11, and 16% of overweight people became obese.^14,15^ The study helps to identify how individuals’ BMI categories change within 5 years and whether these changes are consistent across populations. Boys are more likely to become obese than girls, and the chance of obesity is higher in urban areas than in rural areas. The gender imbalance [35% of boys and 22% of girls (age = 6) were obese at baseline in 2013] may be representative of the obesity distribution among preschool children. The patterns of transitions among BMI categories observed in our study are consistent with those reported in previous studies^8,16^ that also found that nearly 50% of participants had been overweight and 75% had been above the 70th percentile for body-mass index at baseline. Personalized interventions in high-risk primary school children may effectively reduce the incidence of obesity during puberty and adulthood, and some cohort studies on adolescent obesity have also confirmed this view.^17–19^ Preventive and proactive interventions would likely be highly cost-effective.

To our knowledge, this is the first model-based longitudinal study in China to quantify the predictive mechanism linking the predictors to obesity incidence 5 years later, and baseline height appears to be a significant predictor. In the study, we used data collected at a 5-year interval and assessed whether participants were obese at the time of observation. Our study benefits from efficient statistical models and algorithms and 70% students are correctly predicted [higher than another study^20^ (55–60%)]. The predicted obesity rate in 2023 indicates that the childhood obesity epidemic may worsen and require prompt prevention. Obesity rates among older boys and girls, as well as rural students, were predicted to be higher in 2023 than 2018. We expect that obesity prevention efforts for younger children and children in rural areas may be effective for these children who are most likely to become obese during childhood and adolescence.

However, our study is not without limitations. The Chinese national standard only applies to children in China. The criteria for determining overweight and obesity may differ from those of the WHO; thus, the direct application of the results of this study in other countries may result in bias. Our study followed-up the cohort only after 5 years and thus lacked information before elementary school enrollment and after high school graduation. This model is only valid for primary school students. The established predictive model using survey data from 2013 and 2018 may be subject to modifications in predictions for different years (e.g., 2023) due to the temporary evolution of the model parameters and other unmeasured confounding factors. We make the independence assumption between individual students in our study simply because no clustering effects (e.g., among different schools) or spatial correlations (e.g., among different locations) can be conceived and incorporating these factors will take time in our future study. A small proportion of students are missing due to issues irrelevant to our study and we do not involve missing mechanism into our modeling for the time being. However, the procedures we propose and the results we obtained are valuable for studying trends in the prevalence of childhood obesity and proposing interventions in Shandong Province and even other parts of China.

Our study provides support for the strong prospective association of baseline BMI and height with future BMI category. Other studies have shown that children with healthy behavioral habits have lower BMI at follow-up and a lower risk of overweight and obesity.^21^ Therefore, our future research will investigate the factors that influence BMI and determine the relevant factors for weight gain as the focus of intervention. Furthermore, long-term longitudinal studies are warranted to monitor trends in BMI changes and provide more data to validate the predictive model. Finally, this study can provide more epidemiological information for improving childhood and adolescent obesity surveillance and insights into the nature of the obesity epidemic; however, we could not completely rule out residual confounding factors due to unmeasured potential confounders.

## Data Availability

The data used during the current study are not publicly available but are available from the corresponding author upon reasonable request.
